# Clinical neuropsychologists need a standard preliminary observational examination of cognitive functions

**DOI:** 10.3389/fpsyg.2013.00314

**Published:** 2013-05-30

**Authors:** Carlo Abbate, Pietro D. Trimarchi

**Affiliations:** ^1^Unità Operativa Complessa di Geriatria, Ospedale Maggiore Policlinico, Fondazione IRCCS Ca' GrandaMilan, Italy; ^2^Unità Valutazione Alzheimer, Istituto Palazzolo, Fondazione Don Carlo GnocchiMilan, Italy

## Introduction

The clinical neuropsychologist is the specialist who makes diagnosis of cognitive function impairments and infers the relationship between cognitive impairments and the underlying brain damage. Clinical interview with the patient and psychometric tests are the two fundamental instruments available to the neuropsychologist. Certainly, psychometric tests are the core method, however, many clinicians underline the importance of carrying out a preliminary clinical interview during a neuropsychological assessment, talking with the patient and observing her/his behavior in order to identify signs and symptoms of cognitive impairments (Lezak et al., 2004; Hebben and Milberg, 2009). Doing so, expert observers can identify deficits of many cognitive domains (Evans, 2003). Although this clinical practice is commonly adopted, it remains poorly elucidated and it is usually described under vague terms like “informal examination.” Indeed, there is not a standard procedure utilized to observe, register, quantify, interpret (i.e., what cognitive domain is impaired when we observe a specific qualitative data), and report cognitive data detected at the stage of the preliminary interview.

In the present paper we put forward the proposal for a standard version of the preliminary observational evaluation of cognitive functions for neuropsychologists, which we simply label preliminary Neuropsychological Examination (preliminary NPE), in parallel to the standard clinical Neurological Examination (NE).

## Theoretical framework

Broadly speaking, one can found a strong analogy between the clinical practice of a neurologist and the clinical practice of a neuropsychologist. The former concerns the observation of spontaneous signs and symptoms of a neurological dysfunction, both positive (i.e., tremors) and negative (i.e., amimic facies). The latter involves the observation of spontaneous signs and symptoms of a neuropsychological dysfunction, both positive (i.e., confabulations) and negative (i.e., anomic pause). During the NE, the neurologist stimulates patient's motor responses by means of some brief and simple standard actions (i.e., grasping reflex). During the preliminary NPE, the neuropsychologist also provokes patient's behavioral responses by means of some brief and simple questions (i.e., questions about temporal orientation).

In line with this analogy, we think that the clinical neuropsychologist has to more explicitly assume, for the mind examination, the clinical method used in the standard medical practice. The clinical method consists of a sequential series of steps: (1) elicitation of clinical facts, (2) interpretation of signs and symptoms in term of anatomy and physiology, (3) clinical diagnosis, (4) anatomical diagnosis, and (5) pathological and etiological diagnosis (Ropper and Brown, 2005).

In terms of cognitive assessment, the first step (elicitation of clinical facts) aims at collecting symptoms of cognitive dysfunctions referred by the caregiver and complaints reported by patient. The attempt is also to register the signs of cognitive dysfunctions from preliminary NPEs and neuropsychological tests. The second step (interpretation of signs and symptoms in term of anatomy and physiology) aims at interpreting signs and symptoms of the cognitive dysfunction in terms of a standard taxonomy of the cognitive functions (i.e., a functional “anatomy” of the cognitive system) as provided by the modular theory (Marr, 1982; Fodor, 1983) and in terms of processes inside a definite cognitive domain (“physiology” of the cognitive system). Models of normal cognitive processing elaborated by the contemporary cognitive neuropsychology (e.g., the Executive functions model proposed by Shallice) should be the reference for this second step. Considering the third step, a neuropsychologist could disclose a clinical diagnosis when the characteristic cluster of symptoms and signs of cognitive dysfunction are recognized (i.e., pure retrograde amnesia: selective failure to recall past information without associated difficulties in learning new information and without a more diffuse cognitive decline). An accurate cognitive clinical diagnosis offers a fundamental contribution to the successive steps of anatomical and pathological diagnosis.

## Definition, purpose, and characteristics

The preliminary NPE is a systematic collection of cognitive data based on the observation of a patient behavior during the interaction with the clinician in the context of a preliminary interview. It aims at obtaining a concise description of the patient's current cognitive state.

The preliminary NPE has a structured and flexible form with a specific number of cognitive domains which have been examined. A mixed procedure which includes spontaneous and induced (by questions or brief tests) behavioral responses could be adopted. Preliminary NPE can be carried out in the form of an informal enquiry, using a combination of open questions (i.e., asking the patient for the history of her/his illness) and closed questions (i.e., temporal orientation test) to probe the patient's cognitive status. The administration has to be brief (no more than fifteen-30 min).

In the standard medical practice a clinical cognitive examination is included in the Mental Status Examination (MSE) (Trzepacz and Baker, 1993; Strub and Black, 2000). However, we do not consider the MSE a fully appropriate tool for neuropsychologists. Firstly, because the MSE includes many aspects of the mental functioning which do not directly concern cognitive domain (i.e., mood, thought form and content, etc.). Secondly, among the wide range of mental aspects included in the MSE, cognitive functions (i.e., orientation, speech and language, attention, memory, etc.) are usually not investigated enough and this approach provides a cognitive evaluation that is too superficial to be adopted by clinical neuropsychologists. On the other hand, it is also true that psychological and/or psychiatric symptoms are not uncommon in patients suffering from neuropsychological syndromes and MSE is a useful clinical tool to detect them. Taken together, these considerations suggest that NPE and MSE could be considered complementary tools.

Preliminary NPE mainly depends on the knowledge of the clinician about neuropsychological signs and symptoms rather than on the available normative data based on the performance of healthy controls, as requested by psychometric tests. Thus, a skilled knowledge of the behavioral expression of cognitive dysfunctions (Table 1) is the main prerequisite for the administration of the preliminary NPE.

**Table 1:** **Possible signs and symptoms of some cognitive impairments, detectable with the preliminary Neuropsychological Examination (NPE)**.

**Cognitive domain**	**Neuropsychological disorder**		**Signs and symptoms**
Attention	Selective attention impairments		Internal and external distractibility, attentive captures.
	Vigilance-sustained attention impairments		Drowsiness, attentive fluctuations.
Memory	Anterograde amnesia		Oblivion of recent information, perseveration in discourse.
	Retrograde amnesia		Oblivion of past information on recalling autobiographical data, job information, and the medical history.
Pre-frontal functions	Dysexecutive disorders		Poor or diminished insight, lack of the theme of discourse, perseveration of speech or action components, simplified or confused mental tracking or reasoning.
	Adynamic syndrome		Reduction of verbal or behavioral initiative, mutism, lack of spontaneity, diminished empathy.
	Dysinhibited behavior		Iperorality, jocularity, social inappropriateness, hyperactivity, diminished emotional control.
Speech and language	Aphasia	Dysfluency	Effortful articulation, speech lacking of normal prosodic variations, reduced speech output.
		Phonemic deficit	Phonemic paraphasia, conduites d'approche.
		Syntactic deficit	Simplified syntactic clauses, telegraphic speech output, omission/substitution morphological endings.
		Lexical-semantic deficit	Word-finding problems, circumlocutions, semantic paraphasia.
		Comprehension deficit	Answers are inconsistent with questions, necessity to repeat a same question in absence of hearing loss.
Visuospatial abilities	Topographical disorientation		Impaired orientation in hospital unit.
	Neglect	Motor	Patient does not utilize a limb, which adopts a passive, anomalous posture.
		Personal	Lateralized dressing apraxia: e.g., arm of patient's glasses are misplaced upon one of his ears.
		Extrapersonal	Head and eyes exploration movements are limited, shortened or defective toward a region of space, patient does not answer questions when prompted from a side of his space.
Other cognitive functions	Optic ataxia		Misreaching errors trying to grasp objects on the table.
	Gaze apraxia		Abnormal eye and head movements during attempted changes in gaze, fixed gaze, etc.
	Alien hand syndrome		Anomalous posture of arms, levitation, intermanual conflict.
	Ambient dependence syndrome		Echopraxis, echolalia, imitation behavior, utilization behavior.
	Frontal memory disorders		Confabulations, interference, errors concerning the source of the memories.

Preliminary NPE offers to the clinician a set of empirical observations and objective descriptions of behaviors which are related to neuropsychological dysfunctions. It is not a questionnaire or a rating scale compiled by patients or care givers and it is not a screening test like the Mini Mental State Examination (MMSE) (Folstein et al., 1975), even if some brief tasks are included. These tools do not provide reliable and detailed information about the impairment of different cognitive domains, so they cannot represent a substitute for the preliminary NPE.

Preliminary NPE concerns the mind and not the brain. The aim of a neuropsychologist is to recognize/identify a partial or a complete neuropsychological disorder by detecting and correctly classifying its behavioral appearance (mind-behavior relationship). Neuropsychological disorders are primarily dysfunctions of the mind processes. Therefore, the outcomes of the preliminary NPE are accurate descriptions of the mind dysfunctions. The underlying brain damage and its relation with the resulting cognitive impairment (brain-mind relationship) are crucially important from a clinical standpoint, nonetheless they do not directly concern the preliminary NPE.

Preliminary NPE is not an all-inclusive, exhaustive and brief exam of all cognitive functions, but it provides information only for some selected cognitive functions (among the most relevant are psychomotor speed, orientation, attention, insight, prefrontal functions, language, and memory). It only includes those cognitive domains for which qualitative observations or brief tests are sufficient to draw valid and reliable conclusions about the impairment or preservation of those domains (i.e., a spontaneous speech examination during a brief conversation is often sufficient to establish the presence of aphasia, vice-versa, a brief sample of spontaneous writing may not be sufficient for recognizing dysgraphia).

Moreover, the preliminary NPE may not be sufficient for detecting a cognitive impairment. It is probable that some patients who have no impairment on the preliminary NPE, may exhibit some deficit at the following neuropsychological test assessment (e.g., Mild Cognitive Impairment patients or Mild Traumatic Brain Injury patients). Preliminary NPE is, in fact, one of the tools involved in the neuropsychological assessment and it should be integrated with an accurate anamnesis of the cognitive impairments taken from relatives and caregivers, a complete survey of the patients' cognitive complaints and both quantitative and qualitative data from psychometric tasks.

## Rationale: “why do we need a standard preliminary-observational NPE?”

Psychometric tests are certainly the core tool of a neuropsychological assessment, nonetheless this method has some limitations. Firstly, there is a certain heterogeneity assumed by the testing procedures (i.e., using different neuropsychological tests to assess the same cognitive function, using different procedures for scoring the same ability, using normative data from different samples or using distinct neuropsychological test batteries for detecting cognitive impairment caused by distinct pathologies). The major consequence of this heterogeneity is that cognitive data become not very comparable and the communication among clinicians most difficult. Secondly, the time spent for neuropsychological evaluation using psychometric tests is usually very extensive (more than 3 h for a comprehensive assessment, see Lezak et al., 2004). Thirdly, there are many occasions in which a neuropsychological evaluation with psychometric tests cannot be performed (i.e., brain damage patient in acute phase) or may be difficult to administer it (i.e., patients with serious sensory impairment, with important behavioral disorders, or with low educational level). Finally, repeatability of test evaluation is limited because of possible learning effect. The preliminary NPE should be not considered a substitute for a test evaluation, however, it has clear advantages in respect of the standard testing procedures. The preliminary NPE is a standard inventory, fixed and unvarying, therefore it is easy to compare results obtained by different patients, between different conditions or repeated follow-up evaluations in a same patient. It is brief and economical, always administrable (i.e., an adapted form of the preliminary NPE can be administered also to patients in minimally conscious state) and much more repeatable than a standard test assessment. Last but not least, results on the preliminary NPE are easy to communicate to other neuropsychologists.

Sometimes, brain damaged patients are not referred to a neuropsychologist because of the limitations of the test assessment discussed above (i.e., it is time consuming, patients in acute phases or with serious sensory defects may be difficult to test, etc.). Adopting a standard preliminary NPE, which is virtually always administrable, could help to augment the number of patients referred to neuropsychology services and it could consequently help to promote the role of neuropsychologists. Moreover, the preliminary NPE allows a neuropsychologist to decide and manage the referral to a subsequent test evaluation, with the evident advantage of ameliorating the appropriateness of the requests for a complete neuropsychological assessment.

A standard preliminary NPE is a useful tool also to teach clinical neuropsychology. Teaching preliminary NPE emphasizes deep, complete, and accurate descriptions on how different neuropsychological syndromes manifest themselves in the patients' behavior. This list of signs and symptoms are progressively learned by students and, in the course of time, it becomes a mental check list in their minds.

## Conclusion

The aim of this paper is to open a discussion on the need of a standard preliminary observational examination of the cognitive functioning designed for neuropsychologists. Actually, we do not claim to be able to recommend a definite form of the preliminary NPE. A discussion concerning the theoretical framework and the assumptions of a preliminary NPE is needed. It should be established how many and which cognitive domains have to be included in the preliminary NPE. Signs and symptoms of cognitive dysfunction for each cognitive domain should be clearly identified and defined. The variants of the same sign should be considered (i.e., continuous, recurrent, and stuck in set perseverations); the intensity of a sign should be quantified (i.e., minimal, mild, moderate, severe) and its relation with a definite neuropsychological disorder should be validated (e.g., confabulations and prefrontal functions disorders). The issue on how to interpret qualitative data obtained from “informal exams” is surely not new in neuropsychology (see Lezak et al., 2004). But, we think that it is time for a novel, more “formal” and scientific approach to qualitative data. In this frame of reference, we could start from terminology, by substituting the term “qualitative data” with the terms “signs” and “symptoms.” After all, clinical neuropsychologists are indeed clinicians, but how can we be clinicians if we have not a standard preliminary observational examination of cognitive functions?
